# A Short Dietary Screener Captures Food Items and Dietary Patterns That Associate With Inflammation in Inflammatory Bowel Disease

**DOI:** 10.1093/crocol/otaf052

**Published:** 2025-11-05

**Authors:** Gala M Godoy-Brewer, Chunsu Jiang, Nidah S Khakoo, Alejandro Mantero, Yalda Zarnegarnia, Maria A Quintero, Luis Garces, Maria T Abreu, Amar R Deshpande, David H Kerman, Siobhan Proksell, Yasmin Mossavar-Rahmani, Oriana M Damas

**Affiliations:** Department of Medicine, University of Miami School of Medicine, Miami, FL, United States; Crohn’s and Colitis Center, Division of Digestive Health and Liver Diseases, University of Miami Miller School of Medicine, University of Miami, Miami, FL, United States; Department of Medicine, University of Miami School of Medicine, Miami, FL, United States; Department of Biostatistics, University of Miami Miller School of Medicine, Miami, FL, United States; Department of Biostatistics, University of Miami Miller School of Medicine, Miami, FL, United States; Crohn’s and Colitis Center, Division of Digestive Health and Liver Diseases, University of Miami Miller School of Medicine, University of Miami, Miami, FL, United States; Crohn’s and Colitis Center, Division of Digestive Health and Liver Diseases, University of Miami Miller School of Medicine, University of Miami, Miami, FL, United States; Crohn’s and Colitis Center, Division of Digestive Health and Liver Diseases, University of Miami Miller School of Medicine, University of Miami, Miami, FL, United States; Crohn’s and Colitis Center, Division of Digestive Health and Liver Diseases, University of Miami Miller School of Medicine, University of Miami, Miami, FL, United States; Crohn’s and Colitis Center, Division of Digestive Health and Liver Diseases, University of Miami Miller School of Medicine, University of Miami, Miami, FL, United States; Crohn’s and Colitis Center, Division of Digestive Health and Liver Diseases, University of Miami Miller School of Medicine, University of Miami, Miami, FL, United States; Division of Health Behavior Research and Implementation Science, Department of Epidemiology and Population Health, Albert Einstein College of Medicine, Bronx, NY, United States; Crohn’s and Colitis Center, Division of Digestive Health and Liver Diseases, University of Miami Miller School of Medicine, University of Miami, Miami, FL, United States

**Keywords:** inflammatory bowel disease, Dietary Screener Questionnaire, diet, C-reactive protein, fecal calprotectin

## Abstract

**Objectives:**

Diet is important in inflammatory bowel disease (IBD) management, but dietary assessments for clinic use are lengthy and not readily interpretable. The aim of our study was to assess the ability of a short Dietary Screener Questionnaire (DSQ) to capture food items and dietary patterns that are associated with IBD-related inflammation.

**Methods:**

We performed a retrospective study in adult patients with IBD who completed the DSQ from January 2019 to June 2021. Biomarkers C-reactive protein (CRP) and fecal calprotectin (Fecal Cal) were captured within 60 days of DSQ completion. General estimating equations examined relationships between food items and CRP or Fecal Cal. Machine learning was performed to develop dietary patterns.

**Results:**

A total of 1067 patients completed the DSQ, and 577 had biochemical data; 40% were Hispanic. Several food items on the DSQ were associated with inflammatory markers on repeated measures. For instance, red meat [OR: 2.57 (1.19-5.56), *P* = .02], pastry desserts [OR: 2.13 (1.04-4.36), *P* = .04], and beans [OR: 4.2 (1.23-12.51), *P* = .02] were associated with higher inflammation (CRP). High vegetable intake [OR: 0.44 (0.22-0.88), *P* = .02] and baked whole grain goods [OR: 0.15 (0.03-0.67), *P* = .014] were associated with lower inflammatory markers (Fecal Cal). A dietary pattern defined by the lowest fruit and vegetable intake had the highest CRP levels (*P* < .001).

**Conclusions:**

The DSQ is a short dietary screener that can identify food items that associate with inflammation in IBD. Our findings suggest that the DSQ is a feasible tool for use in clinical practice to assess, guide, and track dietary recommendations in a practical way.

## Introduction

Inflammatory bowel disease (IBD) has evolved into a global disease as the incidence increases in developing nations across the world.^1^ In the United States and Europe alone, over 3.5 million people have IBD.^1^ Inflammatory bowel disease is a chronic immune-mediated disease occurring from a combination of genetic susceptibility and environmental exposures, including diet.^2–5^ Several population-based studies now show compelling associations between a Western diet, composed of a diet high in animal protein and ultra-processed foods and low in fruits and vegetables, and increased risk of IBD.^6–8^ Beyond epidemiologic studies implicating the role of diet in IBD development, several observational studies and diet trials show that prudent diets, consisting of high intake of vegetable fiber and fruits, and low intake of animal protein and processed foods, are associated with improvements in clinical disease activity and prevention of relapse in IBD.^9–16^ However, most diet studies have been either small in sample size, have examined disease activity subjectively using clinical symptoms, or have ascertained diet using one-time assessments.

Another challenge in nutrition research is that traditional dietary assessments, including food frequency questionnaires and 24-hour dietary recalls, are lengthy, taking close to an hour to complete, and are impractical in clinical practice. With increased recognition that diet is an important modifier of disease activity and a general lack of access to dietitians for most patients with IBD, we need to develop better strategies to assess patients’ dietary habits at the time of a clinic visit. A dietary screener is a short questionnaire used to assess diet on a limited number of foods and beverages over a specific period.^17^^,^^18^ Dietary screeners querying intake of relevant foods that associate with gut inflammation are ideal for clinical practice, but there is no evidence that existing available screeners could be used in patients with IBD, nor that these screeners can capture foods that correlate with gut inflammation. We hypothesized that a dietary screener could capture sufficient information to permit identification of patients with IBD with increased or decreased risk of intestinal inflammation. To address this hypothesis, we administered the National Health and Nutritional Examination Survey (NHANES) Dietary Screener Questionnaire (DSQ) to a large cohort of patients with IBD. The DSQ is a 26-item questionnaire that takes approximately 10-15 minutes to complete and has many of the foods previously identified in the IBD literature implicated in gut inflammation (or implicated in symptoms).^17^^,^^18^ In our study, we examined whether using a short dietary screener could identify specific food items or a composite dietary pattern that associates with biochemical markers of inflammation (C-reactive protein [CRP] or fecal calprotectin [Fecal Cal]). We leveraged repeated measures of diet intake and markers of inflammation for each patient to better understand the causal relationship between diet intake and ongoing inflammation.

## Methods

### Study design and patient population

**Figure 1.**
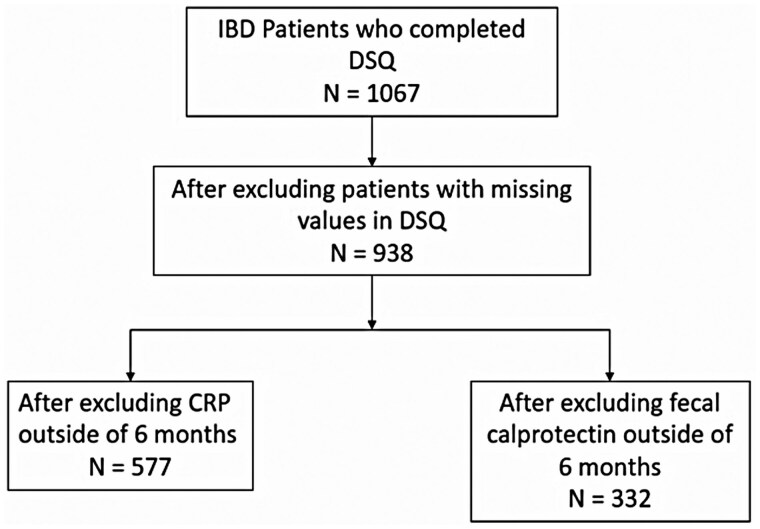
Flow diagram depicting the inclusion and exclusion criteria for patient selection in the study.

This was a retrospective study of adult patients (age ≥18 years) with Crohn’s disease (CD), ulcerative colitis (UC), or IBD unclassified (IBDU) who were seen in an IBD referral clinic in Miami, FL, from January 2019 to June 2021. Patients seen in our IBD clinic who had a primary International Classification of Diseases (ICD) diagnosis of UC, CD, or indeterminate colitis, the ICD code for IBDU, were included in the study. To be seen at our IBD clinic, patients must have had an established or suspected diagnosis of IBD, but to ensure an IBD diagnosis, an International Classification of Diseases (ICD-10) diagnosis check was also performed to ensure diagnostic accuracy. Since 2019, our IBD clinic has routinely collected patients’ diet data via electronic medical record (EMR) as part of the electronic check-in visit checklist. Patients who completed the 26-item DSQ were included in the study. Multiple DSQs captured for each patient for different clinic visits were recorded to provide longitudinal data across the 2 years of the study. We excluded patients who were missing questions on the DSQ. Patients who did not have a CRP or Fecal Cal within 2 months after DSQ completion were excluded from the analysis (Figure 1). To account for the selection bias that those completing the DSQ had lower or higher inflammatory markers than patients who did not complete the DSQ, we examined CRP in patients attending the IBD clinic who did not have a completed DSQ in the same period of observation. Clinical information, including age, sex, race, ethnicity, body mass index (BMI), smoking history, current medication use, disease type (UC, CD, or IBDU), and surgical history, was obtained from the EMR.

### Dietary Screener Questionnaire

The DSQ is a diet questionnaire created for the NHANES in 2009-2010 in place of lengthier assessments such as food frequency questionnaires.^18–20^ The DSQ queries the intake of 26 selected foods in the past month. Foods queried include intake of red meat, sodas, fruits, and vegetables (Table 1). Food items were chosen to be part of the screener because these dietary factors are of interest to the Dietary Guidelines for Americans.^20^ We chose this screener for our IBD cohort because selected food items were associated with inflammation in previous IBD studies.^21–24^ Unlike other dietary screeners available, the DSQ captures information on red meat and processed meat, which are food items previously reported as pro-inflammatory in IBD.^18–20^ The NCI developed algorithms from the screeners to create 11 predicted dietary factors derived from the 26 selected food items (Table 1). Dietary factors included fiber (in grams), added sugars (in teaspoon [tsp] equivalents), and whole grains (in ounce equivalents) (Table S1). We calculated these predicted dietary factors using regression models, adjusting for age and sex specific median portion sizes. Calculations were done using the NHANES-provided SAS macros.^25^ In prior studies, these predicted daily intakes were validated against comprehensive dietary measures such as the Food Frequency Questionnaires (FFQ) and the Automated Self-administered 24-hour (ASA24) Dietary Assessment Tool with moderate correlation and acceptable agreements.^26^^,^^27^ Comparing the screener to 24-hour recalls, fiber intake, for example, had moderate correlations compared to DSQ (0.54-0.55 for women and 0.52-0.6 for men).^27^

**Table 1.** The Dietary Screener Questionnaire (DSQ) and predicted intakes.

**Question**	**Answers**
**How old are you? **	Age in years
**Are you male or female? **	Male or female
**1. During the past month, did you eat any hot or cold cereals?/During the past month, how often did you eat hot or cold cereals? You can report per day, per week, or per month. **	Per day Per week Per month Do not know
**2. During the past month, did you have any milk (either to drink or on cereal)? Include regular milks, chocolate or other flavored milks, lactose-free milk, buttermilk. Do not include soy milk or small amounts of milk in coffee or tea./During the past month, how often did you have any milk (either to drink or on cereal)? You can report per day, per week, or per month. ** ** 2.2 During the past month, what kind of milk did you usually drink? **	Per day Per week Per month Do not know Whole or regular milk 2% fat or reduced-fat milk 1%, ½%, or low-fat milk Fat-free, skim, or nonfat milk Soy milk Other
**3. During the past month, did you drink any regular soda or pop that contains sugar? Do not include diet soda./During the past month, how often did you drink regular soda or pop? You can report per day, per week, or per month. **	Per day Per week Per month Do not know
**4. During the past month, did you drink any 100% pure fruit juices such as orange, mango, apple, grape, and pineapple juices? Do not include fruit-flavored drinks with added sugar or fruit juice you made at home and added sugar to./During the past month, how often did you drink 100% pure fruit juice? You can report per day, per week, or per month. **	Per day Per week Per month Do not know
**5. During the past month, did you drink any coffee or tea that had sugar or honey added to it? Include coffee and tea you sweetened yourself, and presweetened tea and coffee drinks such as Arizona Iced Tea and Frappuccino. Do not include artificially sweetened coffee or diet tea./During the past month, how often did you drink coffee or tea containing sugar or honey? You can report per day, per week, or per month. **	Per day Per week Per month Do not know
**6. During the past month, did you drink any sweetened fruit drinks, sports or energy drinks, such as Kool-Aid, lemonade, Hi-C, cranberry drink, Gatorade, Red Bull, or Vitamin Water? Include fruit juices you made at home and added sugar to. Do not include diet drinks or artificially sweetened drinks./During the past month, how often did you drink sweetened fruit, sports, or energy drinks? You can report per day, per week, or per month. **	Per day Per week Per month Do not know
**7. During the past month, did you eat any fruit? Include fresh, frozen, or canned fruit. Do not include juices./During the past month, how often did you eat fruit? You can report per day, per week, or per month. **	Per day Per week Per month Do not know
**8. During the past month, did you eat a green leafy or lettuce salad, with or without other vegetables?/During the past month, how often did you eat salad? You can report per day, per week, or per month. **	Per day Per week Per month Do not know
**9. During the past month, did you eat any kind of fried potatoes, including French fries, home fries, or hash brown potatoes?/During the past month, how often did you eat any kind of fried potatoes? You can report per day, per week, or per month. **	Per day Per week Per month Do not know
**10. During the past month, did you eat any other kind of potatoes, such as baked, boiled, mashed potatoes, sweet potatoes, or potato salad?/During the past month, how often did you eat any other kind of potatoes? You can report per day, per week, or per month. **	Per day Per week Per month Do not know
**11. During the past month, did you eat any refried beans, baked beans, beans in soup, pork and beans, or other cooked dried beans? Do not include green beans./During the past month, how often did you eat refried beans, baked beans, beans in soup, pork and beans, or other cooked dried beans? You can report per day, per week, or per month. **	Per day Per week Per month Do not know
**12. During the past month, did you eat any brown rice or other cooked whole grains, such as bulgur, cracked wheat, or millet? Do not include white rice./During the past month, how often did you eat brown rice or other cooked whole grains? You can report per day, per week, or per month. **	Per day Per week Per month Do not know
**13. During the past month, not including green salads, potatoes, and cooked dried beans, did you eat any other vegetables?/During the past month, how often did you eat other vegetables? You can report per day, per week, or per month. **	Per day Per week Per month Do not know
**14. During the past month, did you eat any Mexican-type salsa made with tomato?/During the past month, how often did you have Mexican-type salsa made with tomato? You can report per day, per week, or per month. **	Per day Per week Per month Do not know
**15. During the past month, did you eat any pizza? Include frozen pizza, fast food pizza, and homemade pizza./** **During the past month, how often did you eat pizza? You can report per day, per week, or per month. **	Per day Per week Per month Do not know
**16. During the past month, did you have any tomato sauces such as with spaghetti or noodles or mixed into foods such as lasagna? (Do not count tomato sauce on pizza.)/During the past month, how often did you have tomato sauces? You can report per day, per week, or per month. **	Per day Per week Per month Do not know
**17. During the past month, did you eat any kind of cheese? Include cheese as a snack, cheese on burgers, sandwiches, and cheese in foods such as lasagna, quesadillas, or casseroles. Do not include cheese on pizza./During the past month, how often did you eat any kind of cheese? You can report per day, per week, or per month. **	Per day Per week Per month Do not know
**18. During the past month, did you eat any red meat, such as beef, pork, ham, or sausage? Do not include chicken, turkey, or seafood. Include red meat you had in sandwiches, lasagna, stew, and other mixtures. Red meats may also include veal, lamb, and any lunch meats made with these meats./During the past month, how often did you eat red meat? You can report per day, per week, or per month. **	Per day Per week Per month Do not know
**19. During the past month, did you eat any processed meat, such as bacon, lunch meats, or hot dogs? Include processed meats you had in sandwiches, soups, pizza, casseroles, and other mixtures./During the past month, how often did you eat processed meat? You can report per day, per week, or per month. **	Per day Per week Per month Do not know
**20. During the past month, did you eat any whole grain bread, including toast, rolls, and in sandwiches? Whole grain breads include whole wheat, rye, oatmeal, and pumpernickel. Do not include white bread/During the past month, how often did you eat whole grain bread? You can report per day, per week, or per month. **	Per day Per week Per month Do not know
**21. During the past month, did you eat any chocolate or any other types of candy? Do not include sugar-free candy./During the past month, how often did you eat chocolate or any other types of candy? You can report per day, per week, or per month. **	Per day Per week Per month Do not know
**22. During the past month, did you eat any doughnuts, sweet rolls, Danish, muffins, (pan dulce), or Pop-Tarts? Do not include sugar-free items./During the past month, how often did you eat doughnuts, sweet rolls, Danish, muffins, (pan dulce), or Pop-Tarts? You can report per day, per week, or per month **	Per day Per week Per month Do not know
**23. During the past month, did you eat any cookies, cake, pie, or brownies? Do not include sugar-free kinds./During the past month, how often did you eat cookies, cake, pie, or brownies? You can report per day, per week, or per month. **	Per day Per week Per month Do not know
**24. During the past month, did you eat any ice cream or other frozen desserts? Do not include sugar-free kinds./During the past month, how often did you eat ice cream or other frozen desserts? You can report per day, per week, or per month. **	Per day Per week Per month Do not know
**25. During the past month, did you eat any popcorn?/During the past month, how often did you eat popcorn? You can report per day, per week, or per month. **	Per day Per week Per month Do not know
**Predicted intakes: ** **1. Predicted intake of fiber (g) per day** **2. Predicted intake of calcium (mg) per day** **3. Predicted intake of whole grains (ounce equivalents) per day** **4. Predicted intake of total added sugars (tsp equivalents) per day** **5. Predicted intake of dairy (cup equivalents) per day** **6. Predicted intake of fruits and vegetables, including legumes and French fries (cup equivalents) per day** **7. Predicted intake of vegetables, including legumes and French fries (cup equivalents) per day** **8. Predicted intake of fruits and vegetables, including legumes and excluding French fries (cup equivalents) per day** **9. Predicted intake of vegetables, including legumes and excluding French fries (cup equivalents) per day** **10. Predicted intake of fruits (cup equivalents) per day** **11. Predicted intake of added sugars from sugar-sweetened beverages (tsp equivalents) per day**	

### Independent variables

Our independent variable was dietary intake, which we examined using 2 different approaches. In the first approach, we analyzed the associations between inflammation and the 26 food items in the DSQ. In the second approach, we used the 11 DSQ-derived predicted dietary factors to create dietary clusters and examined their associations with inflammation.

### Outcomes

Our primary outcome was the presence of inflammation as determined by serological markers of inflammation, CRP, and Fecal Cal performed after DSQ completion, within 60 days. We captured repeated measures of diet and markers over time. C-reactive protein assay examined was noncardiac (we excluded high-sensitivity CRP). To minimize heterogeneity of abnormal cut-off limits by laboratory type, we converted CRP into binary variables using laboratory upper limits of normal. Similarly, Fecal Cal was treated as a binary variable using a conservative number greater than 250 µg/mg as cut-off for abnormal.^28^^,^^29^ As a secondary outcome, we examined the stability of the 11 predicted diet factors over time using visual inspection of spaghetti plots.

### Statistical analyses

Clinical and demographic variables were examined using standard measures to check for measures of central tendency and outliers. Current age and BMI were analyzed as continuous variables. Gender, ethnicity (Hispanic vs non-Hispanic), race (White vs non-White), smoking status (smoker vs non-smoker), IBD-related abdominal surgical history, and current medication used were dichotomized into binary variables. We categorized medications by class-type (5-ASA, steroids, immunomodulators, and advanced therapies). For categorical variables, we examined associations using the Chi-square. For continuous variables (age and BMI), we used Student’s *t*-tests. We next examined the relationship between diet independent variables and CRP or Fecal Cal. We initially analyzed associations examining patients together as a group (IBD) and by IBD subtype (UC, CD, and IBDU). After initial analyses, we focused on examining the relationship by IBD group and not subtype. We found that after incorporation of clinical and demographic covariates, our models were no longer robust by IBD subtype, and on initial unadjusted evaluation by IBD subtype, we could see that the associations by subtype were in the same direction in UC and CD. Covariates included in our regression models included demographic and clinical variables identified as significant on univariate analyses. To examine for a selection bias that patients completing the DSQ had more (or less) inflammation than those who did not complete the DSQ, we compared inflammatory markers between those that had completed and not completed the DSQ survey during the same period of evaluation. We also compared inflammatory markers at clinic visits when patients had not completed the DSQ survey to when DSQ was completed.

### Association between DSQ food items and the primary outcome

We examined associations between diet variables and each biochemical marker of inflammation (CRP and Fecal Cal). We performed multivariable regression models that incorporated all 26 food items listed in Table 1. This allowed us to control for multiple food items and clinical variables simultaneously in our model and avoid univariate analyses for each food item. We did a first-pass analysis evaluating associations in patients with complete DSQ and lab data using multivariable regression analysis.

Further, in patients with repeated diet-CRP measures with at least 2 DSQs, we examined the relationship between food items and biochemical markers of inflammation using generalized estimation equation (GEE) multivariable logistic regression modeling for repeated measures of testing. In GEE models, we adjusted for the following clinical covariates: age, ethnicity (Hispanic vs non-Hispanic), IBD subtype (UC, CD, IBDU), tobacco use, BMI, IBD-abdominal surgeries, and IBD-medication type (including steroids).

### Dietary pattern creation and examination of the relationship between diet patterns and the primary outcome

We created diet clusters using the 11 predicted dietary factors and dietary items (Table 1). Unsupervised machine learning using sidClustering was performed to form diet clusters using DSQ diet measurements. SidClustering is a nonparametric clustering algorithm capable of handling both categorical and continuous variables simultaneously.^30^ The number of clusters was determined via the gap statistic and 100 bootstrap iterations, and the clustering variables were further filtered down by a second classification forest from which the VIMP was extracted; a cutoff was selected by visual inspection. Clusters were then compared to CRP and Fecal Cal using Fisher’s exact test as clinical validation and adjusted for multiple testing using Bonferroni correction methods. Predicted dietary factors were calculated using SAS macros provided by NHANES using the SAS Studio edition. All analyses were performed in R version 4.1.1 and cluster version 2.1.2. The study was approved by the University of Miami Institutional Review Board.

## Results

### Demographics and clinical characteristics

A total of 1067 patients with IBD completed the DSQ prior to their check-in at our IBD clinic; a total of 40% patients seen during the observation period completed the DSQ electronically without any reminders from staff or clinicians. After excluding patients with incomplete DSQs and patients without CRP or Fecal Cal within 60 days of DSQ completion, 577 were included in the analyses (Figure 1). Most of our IBD cohort reported being of White race (88.4%), and 7.3% were Black. A total of 40% self-identified as Hispanic. The mean age was 40.6 (SD 15.0). A total of 350 patients had CD, 209 had UC, and 18 had IBDU. A total of 24.8% of patients had an elevated CRP, and 37.9% of patients had an elevated Fecal Cal within 2 months of the initial DSQ. Complete demographics data are shown in Table 2. A total of 299 participants completed the DSQ twice and had associated inflammatory markers for each diet visit. A total of 106 patients had 3 DSQs, 32 had 4 DSQs, and 18 patients had 5 DSQs over a span of 2 years. A total of 1604 patients with IBD were seen in our clinic but did not complete the DSQ as part of their check-in visit during the same time period.

**Table 2.** Demographic and clinical variables in our IBD cohort of patients with completed DSQ and inflammatory markers.

		Inflammatory bowel disease type	
	Total	Crohn’s disease	Ulcerative colitis
**All, *n* (%)**	577	350 (60.66)	209 (36.22)
**Age, mean (std)**	40.65 (14.95)	40.08 (14.77)	41.41 (15.08)
**Body mass index (BMI), mean (std)**	25.82 (5.63)	25.71 (5.53)	26.95 (5.65)
**Sex (female), *n* (%)**	302 (52.34)	179 (51.14)	111 (53.11)
**Race, *n* (%)**			
** Asian**	11 (1.91)	6 (1.71)	5 (2.39)
** Black**	42 (7.28)	31 (8.86)	11 (5.26)
** More than one race**	6 (1.04)	6 (1.71)	0 (0)
** White**	510 (88.39)	304 (86.86)	188 (89.95)
**Ethnicity, *n* (%)**			
** Hispanic or Latino**	234 (40.55)	135 (38.57)	89 (42.58)
**Tobacco use, *n* (%)**			
** Never**	464 (80.42)	281 (80.29)	166 (79.43)
** Quit**	98 (16.98)	61 (17.43)	37 (17.70)
** Current**	15 (2.60)	8 (2.29)	6 (2.87)
**Medications, *n* (%)**			
** 5-aminosalicylates**	173 (29.98)	47 (13.43)^a^	123 (58.85)
** Immunomodulators**	80 (13.86)	49 (14)	29 (13.88)
** Steroids**	178 (30.85)	91 (26)^a^	81 (38.76)
** Any biologic**	349 (60.49)	245 (70)^a^	98 (46.89)
**No medications for IBD, *n* (%)**	86 (14.90)	56 (16)	23 (11)

For race, missing and unknown are not shown. Student’s *t*-test was used to compare age and BMI between Crohn’s disease and ulcerative colitis. Chi-square test was used for comparison of all other variables (categorical) in this table.

a Denotes statistical significance (*P* < .05) when comparing each row’s variables between patients with Crohn’s disease and ulcerative colitis.

Abbreviations: DSQ, Dietary Screener Questionnaire; IBD, inflammatory bowel disease.

**Figure 2.**
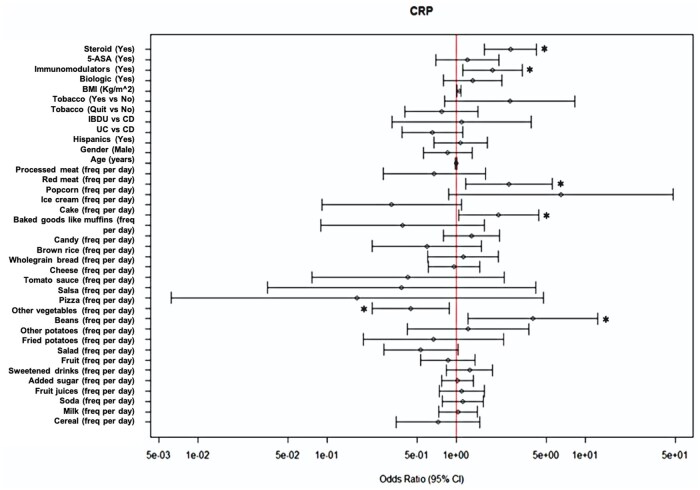
A forest plot identifies an association between medication use and food items with elevated C-reactive protein levels on the dietary screener questionnaire. The vertical line represents an OR = 1. The left side of the line represents a decreased likelihood of elevated C-reactive protein and vice versa. Confidence intervals that do not cross the vertical line are significant and identified with an asterisk.

**Figure 3.**
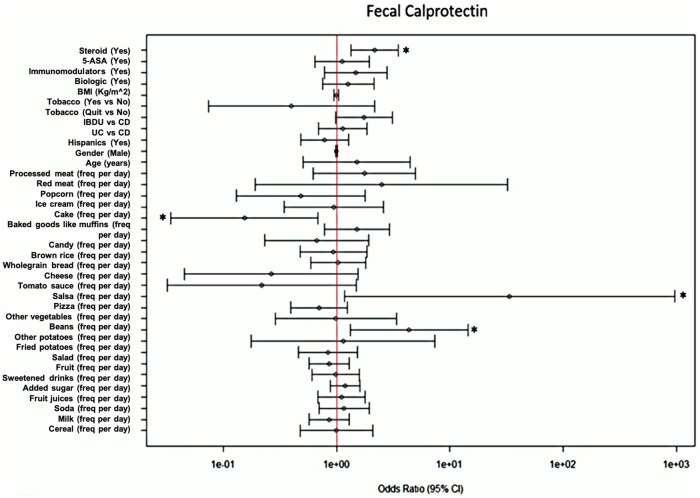
A forest plot identifies associations between medication use and food items with elevated fecal calprotectin levels on the Dietary Screener Questionnaire. The vertical line represents an OR = 1. The left side of the line represents a decreased likelihood of elevated fecal calprotectin and vice versa. Confidence intervals that do not cross the vertical line are significant and identified with an asterisk.

The mean average of DSQs completed for our cohort was 1.88 (SD 1.70, range 1-5). On univariate analyses of demographic and clinical variables with CRP, we found that higher BMI [OR: 1.05 (1.02-1.08), *P *= .002], immunomodulator use [OR: 1.94 (1.26-2.99), *P *= .002], steroid use [OR: 1.92 (1.33-2.77), *P *= .0004], and diagnosis of IBDU (compared to UC and CD) [OR: 2.82 (1.29-6.16), *P *= .009] were positively associated with elevated CRP (Figure S2, Table S4). Steroid use [OR: 1.96 (1.26-3.05), *P *= .002] and diagnosis of UC [OR: 1.61 (1.02-2.56), *P *= .04] were positively associated with elevated Fecal Cal (Figure S3, Table S5). These variables were adjusted for in our regression models. Other sociodemographic and clinical factors (including surgical history) were not associated with elevated markers of inflammation (Figures 2 and 3). When comparing food items and dietary factors by IBD type (UC, CD, IBDU) and ethnicity, we also found similarities in median consumption of each of the food variables examined (data not shown).

We examined CRP in patients attending clinics during this period who had not completed the DSQ surveys (*n* = 1604 patients with IBD). We found that inflammatory markers did not differ between patients completing the DSQ and patients without survey completion (mean CRP value: 0.3 mg/dL vs 0.33 mg/dL, respectively, *P *= .051). We also examined clinical and patient demographic information across these 2 groups and did not identify differences in demographic or clinical characteristics (data not shown). Similarly, for each patient, there were no differences observed between CRP values completed at the time of DSQ completion vs at other visits without DSQ completion (mean CRP 0.24 mg/dL vs 0.23 mg/dL, respectively, *P *= 0.1).

### A short DSQ identifies food items that are associated with lower levels of biochemical inflammation

Using questions derived from the screener, we identified several food items that are associated with CRP and Fecal Cal in multivariable regression models. For instance, we found that high vegetable intake was associated with a lower likelihood of having an elevated CRP [OR: 0.44 (0.22-0.88), *P *= .02] (Table S2). Using Fecal Cal as the outcome, we found that baked goods like muffins were associated with a lower likelihood of having an elevated Fecal Cal [OR: 0.15 (0.03-0.668), *P *= .014] (Table S3). Intake of red meat [OR: 2.57 (1.19-5.56), *P *= .02], pastry desserts [OR: 2.13 (1.04-4.36), *P *= .04], and beans [OR: 4.2 (1.23-12.51), *P *= .02] were associated with high CRP (Figure 2). Further, high intake of potatoes [OR: 4.36 (1.32-14.42), *P *= .02] was associated with an elevated Fecal Cal (Figure 3). Interestingly, consumption of vegetables in the form of salad was not associated with lower levels of CRP or Fecal Cal (Figures 2 and 3). We did not identify associations between other food items and high inflammatory markers in multivariable models, including whole grains (measured as whole grain bread and rice, as well as cereals) (Figures 2 and 3).

### Dietary patterns developed from dietary factors of the DSQ associate with elevated CRP values

**Figure 4.**
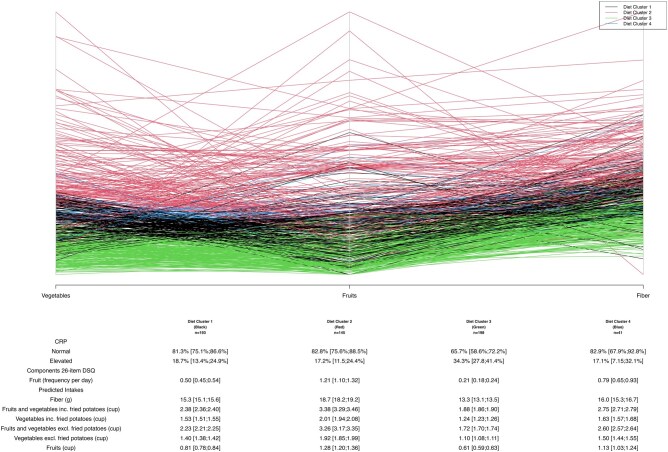
Dietary clusters reveal a dietary pattern composed of the lowest intake of vegetables and fruits is associated with high inflammatory markers. Spaghetti plots of relevant dietary components in the creation of clusters. Each color represents a diet cluster; y-axis represents the daily intake of the variables. The table shows the percentage of patients with elevated CRP or fecal calprotectin in each cluster. Cluster 3, composed of the lowest fruit and vegetable intake, had significantly higher likelihood of having patients with elevated CRP (*P* > .001). Diet variables that were important in cluster determination were included in bold. CRP, C-reactive protein.

Predicted intake of the 11 dietary factors was lower than that recommended by the USDA: including for fiber [15.51 g/day (SD 2.97) vs 34-35 g/day], whole grain [0.64 oz/day (SD 0.34) vs 3.0 oz/day], fruits [0.88 cups/day (SD 0.41) vs 2 cups/day], and vegetables [1.55 cups/day (SD 0.36) vs 2.0 cups/day]. On the other hand, patients consumed more added sugars (15.23 teaspoons/day [SD 4.81] vs <12 teaspoons/day) than the USDA-based recommendations. This pattern was identified consistently on repeated measures. Given the relationship between food items and inflammation, we next asked whether these described a diet pattern. Four dietary clusters were identified in our patients with IBD. We found that the most discerning dietary components in cluster determination were intake of fruits, vegetables, and fiber (Figure 4). Patients whose dietary pattern was cluster 3, composed of the lowest median consumption of daily fruit (0.61 cups per day) and vegetables (1.1 cups/day), were most likely to have elevated CRP levels compared to patients who consumed other dietary patterns. In diet clusters 1, 2, and 4, daily fruit and vegetable median consumption was higher than in cluster 3 and ranged between 0.83-1.30 cups and 1.40-1.90 cups, respectively. A total of 34.3% of patients in cluster 3 had an elevated CRP compared to 18.7% in cluster 1, 17.2% in cluster 2, and 17.1% in cluster 4, *P *= .0005. After Bonferroni correction for multiple comparisons, there was a significant difference in elevated CRP levels identified between cluster 2 and 3, as well as between cluster 1 and 3 (*P *< .01). Cluster 3 consequently had the lowest intake of fiber with a median intake of 13.3 grams daily compared to other clusters with intake ranging from 15.3 to 18.7 grams daily (Figure S6). There was a similar high intake of sugars and added sugars between clusters and a similar intake of red meats and processed meats (Figure 4). Taken together, cluster 3 was the most “unhealthful” dietary pattern, driven by the lowest consumption of fiber compared to other groups but also by similarly high intake of sugars. To assess a patient’s dietary intake over time, we examined patients who had completed at least 2 DSQs (*n* = 299). On visual examination of sequential DSQs, we found that dietary intake of food items and dietary patterns seemed to be similar and appeared stable over time using Spaghetti plots (Figure S1).

## Discussion

To our knowledge, this is the first study to assess whether a broadly available short dietary screener has practical clinical use for patients with IBD. In this study, we tested and confirmed that the DSQ can capture anti- and pro-inflammatory food items in patients with IBD, thereby it can be used to provide personalized nutritional advice for patients. Our survey was administered to a diverse cohort of 1067 patients with IBD as part of the pre-check-in process, also indicating the ease-of-use for implementation of this dietary survey in clinical ­practice. Our dietary results confirm findings previously described in the literature, which show that a low intake of fruits, vegetables, and fiber is associated with elevated inflammatory markers—CRP and fecal Cal.

The DSQ is a 26-item dietary screener that takes approximately 10-15 minutes to complete. Demonstrating ease of use is the fact that around 40% our patients attending the clinic completed the survey electronically as part of the e-check-in process without any additional reminders or mention of the screener during scheduling. The merit of implementing the DSQ screener in clinic is that directed guidance on intake of pro and anti-inflammatory foods can be given by providers or nurses in real time, in a shorter period than other food questionnaires that might be more time-consuming to complete. Although complete nutritional assessments performed by licensed dieticians are ideal, these are not always available to patients with IBD or in GI practices.

To confirm the utility of the DSQ in IBD, we examined foods that were associated with inflammatory markers, and we were able to identify several food items of significance. Leveraging repeated measures of the diet screener and inflammatory markers for each patient, we found that when examining associations between individual dietary food items and CRP, patients with higher daily red meat intake were 2.57× more likely to have an elevated CRP than patients who consumed a lower intake of daily red meat. Similarly, higher daily consumption of pastry desserts like cookies, cakes, and pies was associated with a 2.13× greater risk of elevated CRP after adjusting for BMI, medications including steroid use, surgeries, and smoking status. These findings were in line with prior studies that identify relationships between red meat intake, added sugars, processed foods, and higher risk of disease flare-ups.^21–24^ Further, on repeated measures, we found that patients with greater daily intake of vegetables had lower CRP values than patients with lower intake (*P *= .02). Similarly, we found that higher intake of whole grains was associated with a lower Fecal Cal level compared to those with lower consumption of fiber from whole grains (*P *= .014). The above food items and their respective associations with CRP or Fecal Cal underscore relationships between diet and inflammation that can be captured with the use of this short screener in patients with IBD.

Furthermore, using novel machine-learning clustering methods, we identify dietary clusters that are separated mainly by fiber intake, driven by intake of fruits and vegetables. Based on the dietary clusters, daily median intake of 0.61 cups or less of fruits and 1.11 cups or less of vegetables was associated with higher CRP values. These findings are in line with prior studies showing that high fiber diets derived primarily from vegetables are considered anti-inflammatory in both mouse models and patients with IBD^11^^,^^27^^,^^30–37^. One study found that high intake of fiber by patients with CD but not UC reduced the risk of CD.^9^ In our study, we find a similar beneficial effect of fiber in both UC and CD, and we find that fiber from whole grains is associated with a lower Fecal Cal level as well. Therefore, our findings help to confirm food associations with IBD-related inflammation.

Perhaps secondary to our diverse cohort, which comprised 40% of Hispanics, and differences in cultural behaviors and cooking methods, we identified unexpected associations not previously described in White, non-Hispanic populations. We found that patients who consumed a higher daily intake of cooked beans were 4× more likely to have a high CRP level [OR: 4.2 (1.23-12.51), *P *= .02]. While the confidence intervals were large, and this finding warrants further validation, this finding highlights that food preparation may play just as important a role as the food item itself in deciphering its influence on inflammation and warrants further study. Since a large cohort of our patients was Hispanic, it is possible that preparations of beans were often accompanied by the addition of pork or sausages, as is traditionally performed among patients of Caribbean background.^34^ The DSQ, unlike lengthier dietary questionnaires, does not ask about added oils or added ingredients. For instance, we found that vegetable intake is associated with lower levels of inflammation, but not potatoes or vegetables when consumed in salad form. Our study, therefore, takes the first step toward identifying a dietary tool that is short and feasible for clinical practice and identifies important nuances in the questions asked in the DSQ that warrant further development for IBD practice. Interestingly, the dietary patterns of our patient population closely resembled those reported by Jain et al. in a Northern Texas population that also used the DSQ.^38^

Our study had several strengths. First, we leveraged the dietary screener in a large cohort of patients and employed cautious and robust analytical methods to explore the relationships between food items, dietary factors, and inflammation. We corrected for multiple associations by using Bonferroni correction methods in our analyses and accounted for confounders in our models. We used repeated measures to explore relationships with inflammation and repeated dietary screeners to better gage persistent relationships between both and minimize associations driven by avoidance of foods due to the presence of symptoms. Our cohort of patients was also largely diverse, comprising a large population of Hispanic patients with IBD, which increases the generalizability of our study to a diverse US population. Additionally, we accounted for selection bias to ensure that our cohort completing the DSQ did not differ in severity of inflammation from those who did not complete the DSQ in our clinic. Our study also had several limitations. We administered the dietary screener and captured IBD phenotype information using the EMR, which has inherent limitations of data capture accuracy. However, because we only examined patients followed in our IBD clinic, we were able to eliminate the bias seen in EMR studies that capture IBD patients by ICD-10 code only. A second limitation of our study was that we did not include endoscopic evaluation. While we did not use the gold standard of colonoscopy, CRP and Fecal Cal have acceptable sensitivities and specificities and are commonly used in clinical practice as surrogates of inflammation to guide management.^39^ Future studies should examine the relationship between diet patterns and endoscopic or histologic disease activity. Another limitation is that we did not adjust for physical activity, a factor that could impact the relationship between diet and inflammation. The retrospective nature of our study presents a limitation within itself. When recording dietary patterns over a specific period, we may overlook dietary changes that patients make during symptomatic episodes. However, as mentioned above, overall dietary patterns appeared stable over time. Another retrospective limitation is that not all patients who completed the dietary screeners were included in the analysis—only those with available data on inflammatory markers. One of the strengths of our study is the inclusion of a large Hispanic population; however, the questionnaire has not yet been validated for Hispanic populations. Despite being administered in Spanish, its validity for this demographic remains unconfirmed, which would be an important area for future research.

Lastly, a screener does not assess daily intake but rather makes predictions based on age and sex, sacrificing some accuracy in daily intake for ease of length.^40^ This may in part explain why we did not find any correlations between the individual 11 predicted dietary factors and inflammation. Further, as with any dietary questionnaire, there was recall bias with reported intake.^34^^,^^39^ Dietary information obtained by a DSQ had lower accuracy compared to a full-length food frequency questionnaire with limitations on cooking practices, as mentioned earlier. However, prior studies comparing the screener to multiple 24-hour recalls found correlation coefficients for fiber intake ranging from 0.54 to 0.55 for women and 0.52 to 0.60 for men, which have acceptable correlation.^27^ A similar degree of measurement error between the DSQ and other lengthier dietary assessments also suggests similarities in the degree of information bias present.^20^ One data point we did not include in our study but would be valuable to explore in future research is socioeconomic status, including factors such as zip code, employment, and insurance. Examining these variables could help determine whether socioeconomic status influences dietary patterns and, consequently, inflammation.

In conclusion, our study identifies that a short dietary screener already widely available online has applicability in IBD and identifies food items and dietary patterns that can be pro- and anti-inflammatory. We confirm that dietary patterns with high fiber intake from fruits, vegetables, and whole grains are associated with lower markers of inflammation in both UC and CD, in repeated measures spanning 2 years of observation in some patients. Future studies should focus on building on this dietary screener and testing prospectively whether dietary guidance using the screener can help clinicians improve nutrition education for patients with IBD.

## Supplementary Material

otaf052_Supplementary_Data

## Data Availability

Raw data and code are not publicly available, but available upon request.
