# Supplementary material for: A Short Dietary Screener Captures Food Items and Dietary Patterns That Associate With Inflammation in Inflammatory Bowel Disease
Source: Crohns Colitis 360. 2025 Nov 5;7(4):otaf052. doi: 10.1093/crocol/otaf052 (PMC12623008; doi:10.1093/crocol/otaf052)
Supplement: otaf052_Supplementary_Data [file otaf052_supplementary_data.zip › Supplementary Tables.docx]

**Supplementary figure 1.** Spagetti plots of changes in predicted dietary factors over repeated dietary screener questionnaires.

**
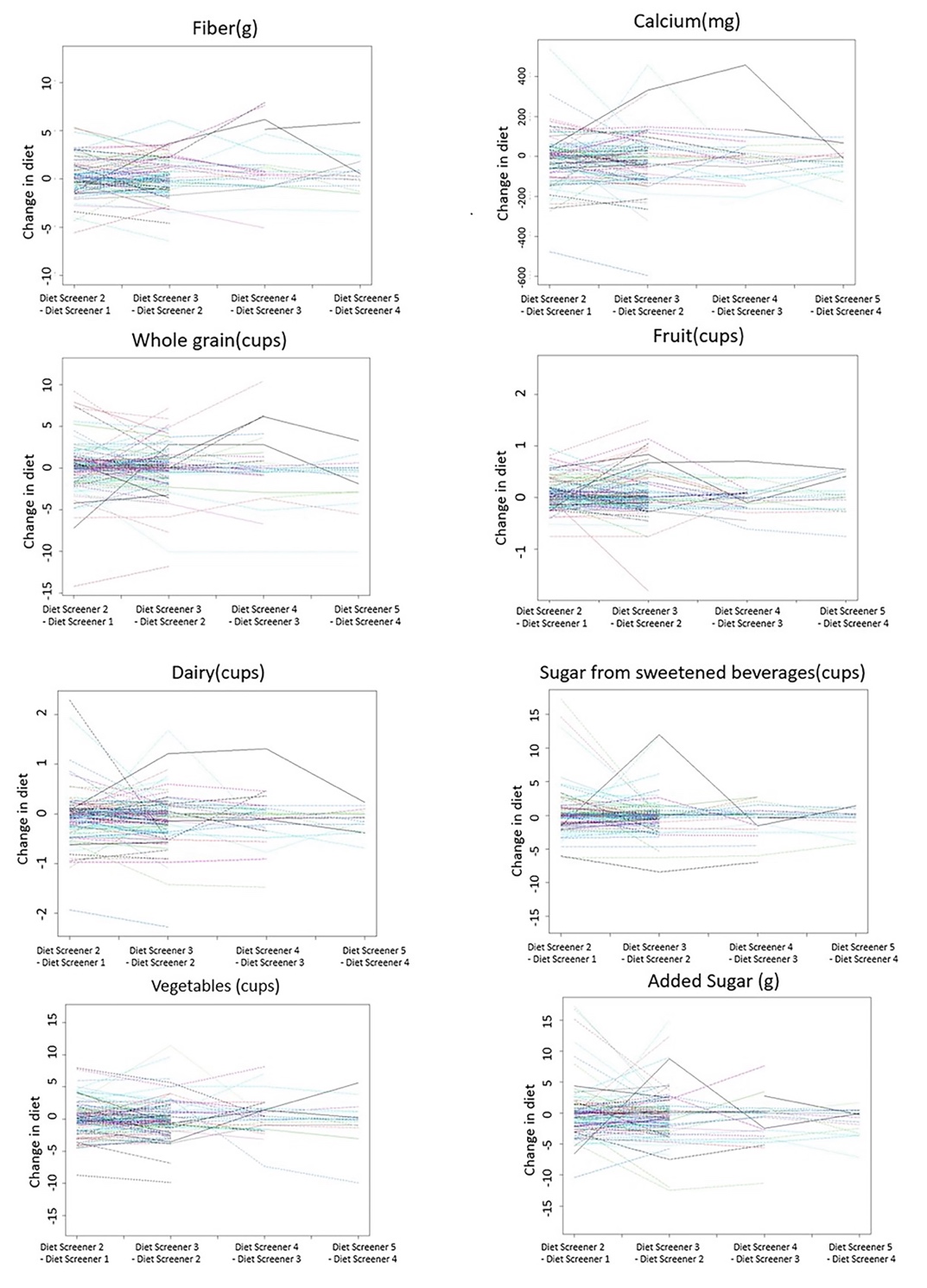
**

Plots were generated from predicted dietary intakes. Each color represents a single patient. Patterns show stable intake over time in terms of fiber, calcium, whole grain, dairy and fruits and vegetable intake. The Y axis notes amount of change in predicted intake of food items, and the X-axis denotes dietary screener questionnaires.

**Supplementary Table 1.** Predicted dietary food intake and food items asked in the dietary screener questionnaire.


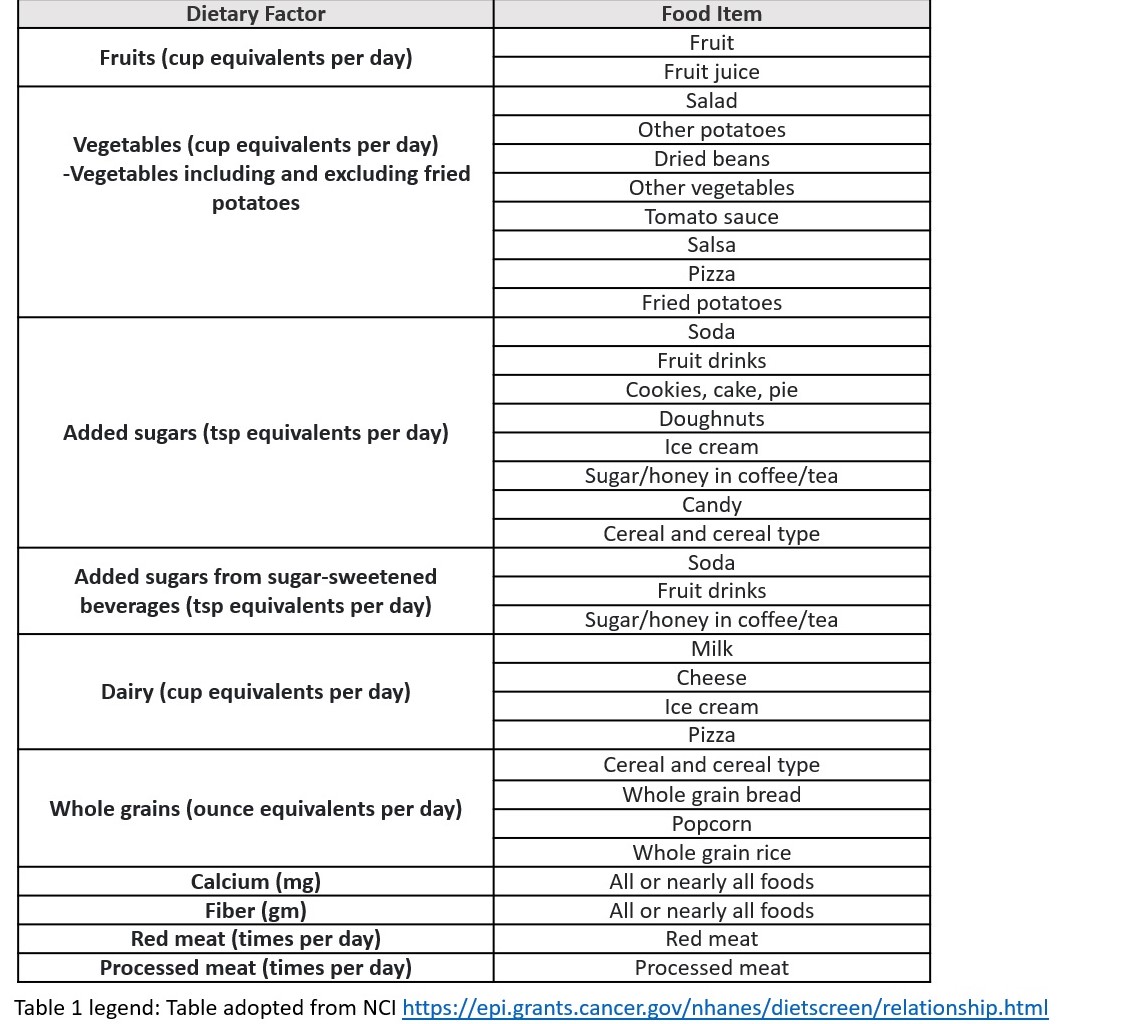


**Supplementary Table 2.** Examination of demographic, clinical and food items with high CRP level.

|  | Estimate | Robust standard error | Robust z-score | OR (Confidence Interval) | *p-*value |
| --- | --- | --- | --- | --- | --- |
| **(Intercept)** | **-2.69** | **0.68** | **-3.98** | **0.07 (0.02 - 0.25)** | **0.0001** |
| Age | 0.00 | 0.01 | 0.04 | 1 (0.98 - 1.02) | 0.9714 |
| Gender (Male vs Female) | -0.15 | 0.22 | -0.68 | 0.86 (0.56 - 1.33) | 0.4947 |
| Ethnicity (Non-Hispanics vs Hispanics) | 0.08 | 0.24 | 0.33 | 1.08 (0.68 - 1.74) | 0.7406 |
| IBD (UC vs CD) | -0.42 | 0.28 | -1.52 | 0.66 (0.38 - 1.13) | 0.1285 |
| IBD (IC vs CD) | 0.10 | 0.63 | 0.15 | 1.1 (0.32 - 3.81) | 0.8808 |
| Tobacco (Quit vs No) | -0.26 | 0.33 | -0.78 | 0.77 (0.4 - 1.48) | 0.4358 |
| tobacco (Yes vs No) | 0.96 | 0.59 | 1.62 | 2.6 (0.82 - 8.31) | 0.1060 |
| **BMI** | **0.05** | **0.02** | **2.86** | **1.05 (1.02 - 1.09)** | **0.0043** |
| Biologics (Yes vs No) | 0.29 | 0.27 | 1.09 | 1.34 (0.79 - 2.25) | 0.2742 |
| **Immunomodulators (Yes vs No)** | **0.65** | **0.27** | **2.40** | **1.91 (1.13 - 3.25)** | **0.0163** |
| 5-ASA (Yes vs No) | 0.20 | 0.29 | 0.69 | 1.22 (0.69 - 2.14) | 0.4884 |
| **Steroids (Yes vs No)** | **0.97** | **0.24** | **4.12** | **2.63 (1.66 - 4.17)** | **<0.0001** |
| Cereal | -0.33 | 0.38 | -0.85 | 0.72 (0.34 - 1.53) | 0.3949 |
| Milk | 0.04 | 0.17 | 0.21 | 1.04 (0.74 - 1.46) | 0.8328 |
| Soda | 0.12 | 0.19 | 0.63 | 1.12 (0.78 - 1.62) | 0.5302 |
| Fruit juice | 0.10 | 0.21 | 0.48 | 1.1 (0.74 - 1.65) | 0.6284 |
| Added sugar to honey/tea | 0.02 | 0.14 | 0.15 | 1.02 (0.77 - 1.35) | 0.8790 |
| Sports/energy drinks | 0.24 | 0.21 | 1.14 | 1.27 (0.84 - 1.92) | 0.2532 |
| Fruits | -0.15 | 0.25 | -0.59 | 0.86 (0.53 - 1.4) | 0.5555 |
| Salad | -0.63 | 0.34 | -1.86 | 0.53 (0.27 - 1.04) | 0.0633 |
| Fried potatoes | -0.40 | 0.64 | -0.64 | 0.67 (0.19 - 2.32) | 0.5253 |
| Other potatoes | 0.21 | 0.55 | 0.38 | 1.23 (0.42 - 3.64) | 0.7042 |
| **Beans** | **1.37** | **0.59** | **2.31** | **3.93 (1.23 - 12.51)** | **0.0206** |
| **Other vegetables** | **-0.81** | **0.35** | **-2.32** | **0.44 (0.22 - 0.88)** | **0.0203** |
| Pizza | -1.76 | 1.69 | -1.04 | 0.17 (0.01 - 4.73) | 0.2974 |
| Salsa | -0.97 | 1.22 | -0.80 | 0.38 (0.03 - 4.13) | 0.4255 |
| Tomato Sauce | -0.86 | 0.87 | -0.98 | 0.42 (0.08 - 2.35) | 0.3255 |
| Cheese | -0.04 | 0.23 | -0.17 | 0.96 (0.61 - 1.52) | 0.8683 |
| Whole grain bread | 0.13 | 0.32 | 0.39 | 1.13 (0.6 - 2.13) | 0.6967 |
| Brown rice | -0.52 | 0.50 | -1.05 | 0.59 (0.22 - 1.57) | 0.2943 |
| Candy/chocolate | 0.27 | 0.26 | 1.06 | 1.31 (0.79 - 2.17) | 0.2892 |
| Doughnuts/pastries | -0.96 | 0.74 | -1.28 | 0.38 (0.09 - 1.66) | 0.1994 |
| Cake/cookies | 0.76 | 0.36 | 2.07 | 2.13 (1.04 - 4.36) | 0.0382 |
| Ice cream | -1.15 | 0.64 | -1.81 | 0.32 (0.09 - 1.1) | 0.0706 |
| Popcorn | 1.87 | 1.02 | 1.83 | 6.46 (0.87 - 47.71) | 0.0674 |
| **Red meat** | **0.94** | **0.39** | **2.39** | **2.57 (1.19 - 5.56)** | **0.0167** |
| Processed meat | -0.39 | 0.46 | -0.84 | 0.68 (0.27 - 1.68) | 0.4005 |

**Supplemental Table 3.** Demographic, clinical and food-item associations with elevated Fecal Calprotectin

|  | Estimate | Robust standard error | Robust z-score | OR (Confidence Interval) | *p*-value |
| --- | --- | --- | --- | --- | --- |
| Intercept | -1.03 | 0.76 | -1.36 | 0.36 (0.08 - 1.57) | 0.1736 |
| Age | 0.00 | 0.01 | -0.39 | 1 (0.98 - 1.01) | 0.6957 |
| Gender (Male vs Female) | -0.24 | 0.25 | -0.99 | 0.78 (0.48 - 1.27) | 0.3237 |
| Ethnicity (Non-Hispanics vs Hispanics) | 0.13 | 0.25 | 0.51 | 1.14 (0.69 - 1.87) | 0.6096 |
| IBD (UC vs CD) | 0.56 | 0.29 | 1.90 | 1.75 (0.98 - 3.1) | 0.0573 |
| IBD (IC vs nonIC) | -0.92 | 0.86 | -1.07 | 0.4 (0.07 - 2.16) | 0.2864 |
| BMI | 0.00 | 0.02 | -0.19 | 1 (0.95 - 1.04) | 0.8492 |
| Biologics (Yes vs No) | 0.24 | 0.27 | 0.89 | 1.27 (0.75 - 2.15) | 0.3728 |
| Immunomodulators (Yes vs No) | 0.39 | 0.33 | 1.19 | 1.48 (0.78 - 2.8) | 0.2321 |
| 5-ASA (Yes vs No) | 0.12 | 0.28 | 0.42 | 1.12 (0.65 - 1.95) | 0.6778 |
| **Steroids (Yes vs No)** | **0.77** | **0.25** | **3.13** | **2.16 (1.33 - 3.49)** | **0.0017** |
| Cereal | 0.00 | 0.38 | 0.00 | 1 (0.48 - 2.09) | 0.9961 |
| Milk | -0.15 | 0.21 | -0.73 | 0.86 (0.57 - 1.29) | 0.4636 |
| Soda | 0.16 | 0.26 | 0.60 | 1.17 (0.7 - 1.95) | 0.5517 |
| Fruit juice | 0.10 | 0.25 | 0.43 | 1.11 (0.68 - 1.8) | 0.6705 |
| Added sugar to honey/tea | 0.17 | 0.15 | 1.10 | 1.18 (0.88 - 1.6) | 0.2722 |
| Sports/energy drinks | -0.02 | 0.25 | -0.07 | 0.98 (0.61 - 1.59) | 0.9464 |
| Fruits | -0.15 | 0.21 | -0.73 | 0.86 (0.57 - 1.29) | 0.4680 |
| Salad | -0.18 | 0.31 | -0.58 | 0.84 (0.46 - 1.53) | 0.5615 |
| Fried potatoes | 0.13 | 0.95 | 0.14 | 1.14 (0.18 - 7.4) | 0.8879 |
| **Other potatoes** | **1.47** | **0.61** | **2.41** | **4.36 (1.32 - 14.42)** | **0.0158** |
| Beans | -0.02 | 0.63 | -0.03 | 0.98 (0.29 - 3.37) | 0.9792 |
| Other vegetables | -0.36 | 0.29 | -1.22 | 0.7 (0.39 - 1.24) | 0.2235 |
| **Pizza** | **3.52** | **1.71** | **2.05** | **33.66 (1.17 - 968.08)** | **0.0402** |
| Salsa | -1.52 | 0.98 | -1.55 | 0.22 (0.03 - 1.49) | 0.1207 |
| Tomato Sauce | -1.33 | 0.90 | -1.47 | 0.27 (0.05 - 1.55) | 0.1407 |
| Cheese | 0.03 | 0.28 | 0.12 | 1.03 (0.59 - 1.81) | 0.9068 |
| Whole grain bread | -0.06 | 0.35 | -0.19 | 0.94 (0.48 - 1.85) | 0.8515 |
| Brown rice | -0.40 | 0.54 | -0.75 | 0.67 (0.23 - 1.92) | 0.4557 |
| Candy/chocolate | 0.41 | 0.34 | 1.22 | 1.51 (0.78 - 2.92) | 0.2243 |
| **Baked muffins** | **-1.87** | **0.76** | **-2.45** | **0.15 (0.03 - 0.69)** | **0.0144** |
| Cake/cookies | -0.05 | 0.51 | -0.10 | 0.95 (0.35 - 2.61) | 0.9228 |
| Ice cream | -0.73 | 0.67 | -1.09 | 0.48 (0.13 - 1.79) | 0.2768 |
| Popcorn | 0.92 | 1.31 | 0.70 | 2.5 (0.19 - 32.41) | 0.4836 |
| Red meat | 0.57 | 0.53 | 1.07 | 1.76 (0.62 - 4.98) | 0.2837 |
| Processed meat | 0.41 | 0.55 | 0.74 | 1.5 (0.51 - 4.45) | 0.4598 |

**Supplementary figure 2.** Multivariate analysis using predicted dietary intake with CRP as outcome.

|  | Estimate | Robust standard error | Robust z-score | OR (Confidence Interval) | *p-*value |
| --- | --- | --- | --- | --- | --- |
| **(Intercept)** | **-1.95** | **0.77** | **-2.55** | **0.14 (0.03 - 0.64)** | **0.0109** |
| Age | -0.01 | 0.01 | -0.94 | 0.99 (0.98 - 1.01) | 0.3475 |
| IBD (UC vs nonUC) | -0.32 | 0.19 | -1.63 | 0.73 (0.5 - 1.07) | 0.1038 |
| **IBD (IC vs nonIC)** | **1.04** | **0.40** | **2.60** | **2.82 (1.29 - 6.16)** | **0.0094** |
| **BMI** | **0.05** | **0.02** | **3.09** | **1.05 (1.02 - 1.08)** | **0.0020** |
| Biologics (Yes vs no) | 0.24 | 0.20 | 1.24 | 1.28 (0.87 - 1.88) | 0.2162 |
| **Immunomodulators (Yes vs no)** | **0.66** | **0.22** | **3.00** | **1.94 (1.26 - 2.99)** | **0.0027** |
| **Steroids (Yes vs no)** | **0.65** | **0.19** | **3.49** | **1.92 (1.33 - 2.77)** | **0.0005** |
| Fiber | 0.04 | 0.08 | 0.49 | 1.04 (0.89 - 1.21) | 0.6216 |
| Calcium | 0.00 | 0.00 | -0.17 | 1 (1 - 1) | 0.8681 |
| Whole grain | -0.45 | 0.31 | -1.42 | 0.64 (0.35 - 1.19) | 0.1562 |
| Added sugar | -0.01 | 0.04 | -0.16 | 0.99 (0.93 - 1.07) | 0.8760 |
| Dairy | 0.01 | 0.46 | 0.03 | 1.01 (0.41 - 2.49) | 0.9769 |
| Fruit and vegetables (excl. fried potatoes) | -0.30 | 1.12 | -0.27 | 0.74 (0.08 - 6.68) | 0.7897 |
| Vegetables (excl. fried potatoes) | -0.39 | 1.18 | -0.33 | 0.68 (0.07 - 6.88) | 0.7432 |
| Fruits | 0.26 | 1.02 | 0.25 | 1.29 (0.17 - 9.57) | 0.8026 |
| Sugar from sweetened beverages | 0.04 | 0.04 | 1.02 | 1.04 (0.96 - 1.13) | 0.3092 |


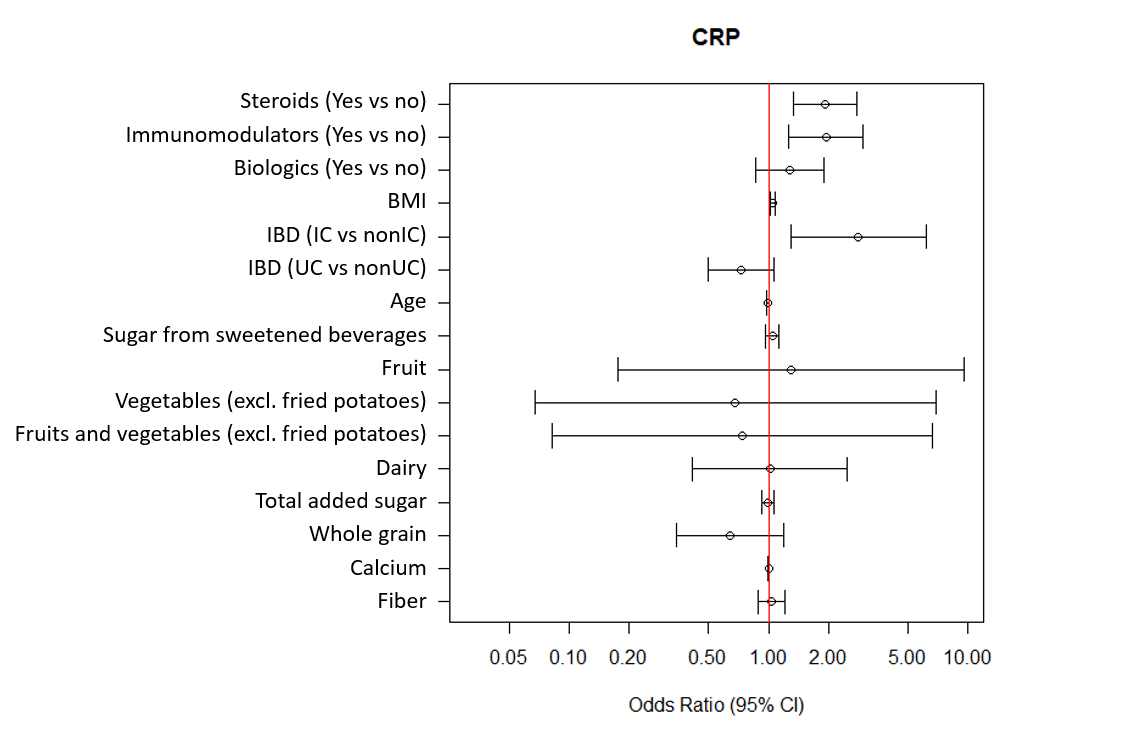


**Supplementary Table 4.** Multivariate analysis using predicted dietary intake with CRP as outcome

**Supplementary figure 3 .** Multivariate analysis using predicted dietary intake with Fecal Calprotectin as
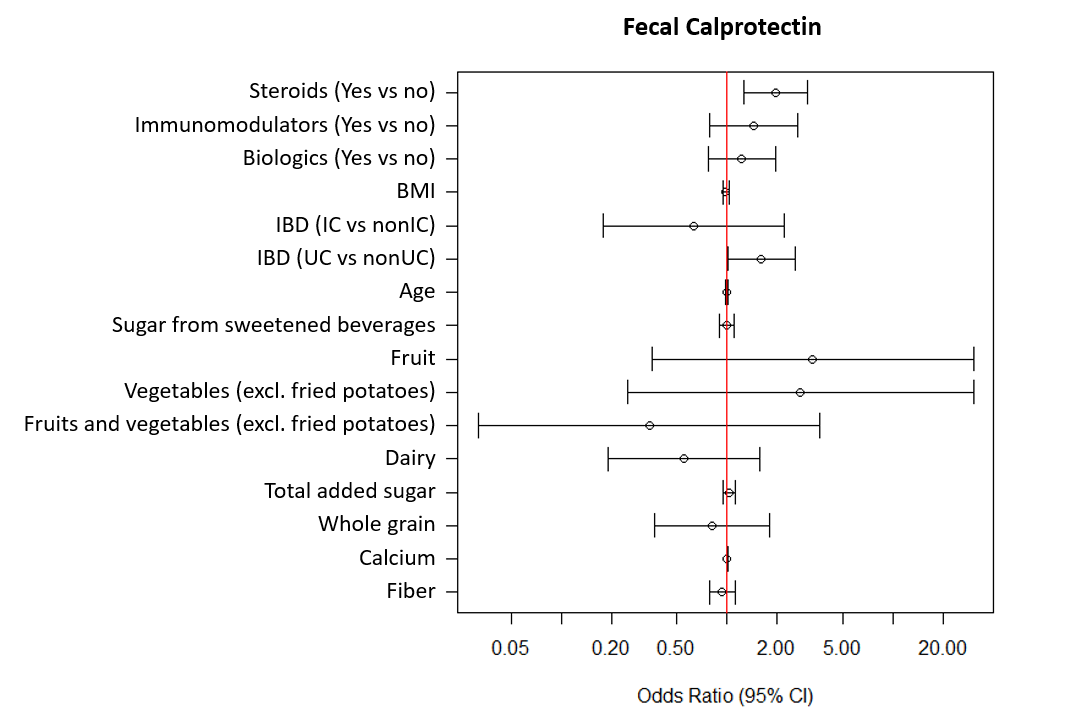
outcome.

**Supplementary Table 5.** Multivariate analysis using predicted dietary intake with Fecal Calprotectin as outcome

|  | Estimate | Robust standard error | Robust z-score | OR (Confidence Interval) | *p-*value |
| --- | --- | --- | --- | --- | --- |
| (Intercept) | -0.08 | 0.94 | -0.08 | 0.93 (0.15 - 5.88) | 0.9343 |
| Age | 0.00 | 0.01 | -0.60 | 1 (0.98 - 1.01) | 0.5496 |
| **IBD (UC vs nonUC)** | **0.48** | **0.24** | **2.03** | **1.61 (1.02 - 2.56)** | **0.0421** |
| IBD (IC vs nonIC) | -0.46 | 0.64 | -0.72 | 0.63 (0.18 - 2.22) | 0.4731 |
| BMI | -0.02 | 0.02 | -0.91 | 0.98 (0.94 - 1.02) | 0.3653 |
| Biologics (Yes vs no) | 0.20 | 0.24 | 0.84 | 1.22 (0.77 - 1.96) | 0.3983 |
| Immunomodulators (Yes vs no) | 0.37 | 0.31 | 1.19 | 1.44 (0.79 - 2.64) | 0.2352 |
| **Steroids (Yes vs no)** | **0.67** | **0.22** | **3.00** | **1.96 (1.26 - 3.05)** | **0.0027** |
| Fiber | -0.07 | 0.09 | -0.80 | 0.93 (0.78 - 1.11) | 0.4264 |
| Calcium | 0.00 | 0.00 | 0.93 | 1 (1 - 1) | 0.3529 |
| Whole grain | -0.21 | 0.40 | -0.51 | 0.81 (0.37 - 1.79) | 0.6076 |
| Added sugar | 0.03 | 0.04 | 0.75 | 1.03 (0.95 - 1.12) | 0.4551 |
| Dairy | -0.60 | 0.54 | -1.12 | 0.55 (0.19 - 1.57) | 0.2617 |
| Fruit and vegetables (excl. fried potatoes) | -1.08 | 1.21 | -0.89 | 0.34 (0.03 - 3.64) | 0.3717 |
| Vegetables (excl. fried potatoes) | 1.02 | 1.23 | 0.83 | 2.77 (0.25 - 30.73) | 0.4068 |
| Fruits | 1.19 | 1.14 | 1.05 | 3.29 (0.36 - 30.49) | 0.2943 |
| Sugar from sweetened beverages | -0.01 | 0.05 | -0.14 | 0.99 (0.9 - 1.1) | 0.8915 |

**Supplementary Table 6.** Summary statistics of both predicted dietary intake and 26-item DSQ of the four dietary clusters and associations with CRP and Fecal Cal.

|  | **Diet Cluster 1**  **(Black)** | **Diet Cluster 2**  **(Red)** | **Diet Cluster 3**  **(Green)** | **Diet Cluster 4**  **(Blue)** |
| --- | --- | --- | --- | --- |
|  | ***N=193*** | ***N=145*** | ***N=198*** | ***N=41*** |
| CRP: |  |  |  |  |
| Normal | 81.3% [75.1%;86.6%] | 82.8% [75.6%;88.5%] | **65.7% [58.6%;72.2%]** | 82.9% [67.9%;92.8%] |
| Elevated | 18.7% [13.4%;24.9%] | 17.2% [11.5%;24.4%] | **34.3% [27.8%;41.4%]** | 17.1% [7.15%;32.1%] |
| Components 26-item DSQ | | | | |
| Cereal (frequency per day) | 0.19 [0.14;0.24] | 0.25 [0.20;0.31] | 0.17 [0.13;0.21] | 0.22 [0.09;0.34] |
| Milk (frequency per day) | 0.41 [0.32;0.51] | 0.52 [0.38;0.65] | 0.41 [0.32;0.50] | 0.54 [0.30;0.77] |
| Soda (frequency per day) | 0.18 [0.12;0.24] | 0.06 [0.04;0.09] | 0.27 [0.19;0.36] | 0.27 [0.06;0.48] |
| Fruit Juices (frequency per day) | 0.24 [0.19;0.29] | 0.34 [0.22;0.47] | 0.13 [0.10;0.16] | 0.60 [0.36;0.85] |
| Added Sugar or Honey to Coffee (frequency per day) | 0.52 [0.43;0.62] | 0.66 [0.47;0.85] | 0.44 [0.35;0.52] | 0.99 [0.59;1.40] |
| Sweetened Drinks (frequency per day) | 0.17 [0.12;0.23] | 0.15 [0.09;0.21] | 0.22 [0.15;0.29] | 0.22 [0.08;0.36] |
| **Fruit** (frequency per day) | **0.50 [0.45;0.54]** | **1.21 [1.10;1.32]** | **0.21 [0.18;0.24]** | **0.79 [0.65;0.93]** |
| Salad (frequency per day) | 0.30 [0.27;0.34] | 0.80 [0.71;0.89] | 0.08 [0.06;0.09] | 0.39 [0.30;0.48] |
| Fried Potatoes (frequency per day) | 0.14 [0.12;0.17] | 0.11 [0.08;0.13] | 0.13 [0.11;0.16] | 0.15 [0.09;0.21] |
| Other Potatoes (frequency per day) | 0.20 [0.17;0.22] | 0.23 [0.19;0.26] | 0.15 [0.13;0.17] | 0.29 [0.21;0.37] |
| Beans (frequency per day) | 0.11 [0.09;0.13] | 0.20 [0.15;0.25] | 0.08 [0.06;0.10] | 0.10 [0.06;0.15] |
| Other Vegetables (frequency per day) | 0.48 [0.42;0.53] | 0.93 [0.83;1.03] | 0.17 [0.14;0.20] | 0.51 [0.36;0.65] |
| Pizza (frequency per day) | 0.06 [0.06;0.07] | 0.07 [0.06;0.08] | 0.08 [0.06;0.09] | 0.07 [0.03;0.11] |
| Salsa (frequency per day) | 0.06 [0.04;0.07] | 0.08 [0.06;0.11] | 0.03 [0.02;0.04] | 0.04 [0.02;0.06] |
| Tomato Sauce (frequency per day) | 0.09 [0.07;0.10] | 0.11 [0.08;0.13] | 0.08 [0.07;0.10] | 0.14 [0.06;0.21] |
| Cheese (frequency per day) | 0.48 [0.41;0.54] | 0.52 [0.44;0.60] | 0.38 [0.32;0.44] | 0.51 [0.36;0.66] |
| Wholegrain Bread (frequency per day) | 0.23 [0.19;0.28] | 0.32 [0.26;0.39] | 0.18 [0.14;0.21] | 0.35 [0.19;0.50] |
| Brown Rice (frequency per day) | 0.13 [0.10;0.16] | 0.18 [0.14;0.22] | 0.08 [0.05;0.12] | 0.14 [0.08;0.20] |
| Chocolate/Candy (frequency per day) | 0.28 [0.23;0.33] | 0.24 [0.19;0.29] | 0.22 [0.18;0.26] | 0.33 [0.19;0.48] |
| Baked Goods, Pastries (frequency per day) | 0.09 [0.06;0.11] | 0.07 [0.04;0.10] | 0.08 [0.06;0.09] | 0.06 [0.03;0.09] |
| Cookies, Cake, Pie (frequency per day) | 0.18 [0.14;0.21] | 0.14 [0.11;0.17] | 0.16 [0.13;0.20] | 0.16 [0.09;0.23] |
| Ice Cream (frequency per day) | 0.12 [0.09;0.14] | 0.10 [0.08;0.13] | 0.11 [0.08;0.14] | 0.13 [0.08;0.19] |
| Popcorn (frequency per day) | 0.04 [0.03;0.06] | 0.04 [0.03;0.06] | 0.02 [0.01;0.02] | 0.04 [0.01;0.07] |
| Red Meat (frequency per day) | 0.28 [0.25;0.31] | 0.27 [0.23;0.32] | 0.26 [0.22;0.31] | 0.27 [0.19;0.34] |
| Processed Meat (frequency per day) | 0.14 [0.12;0.17] | 0.16 [0.12;0.20] | 0.15 [0.12;0.19] | 0.13 [0.07;0.19] |
| Predicted Intakes | | | | |
| **Fiber (g)** | **15.3 [15.1;15.6]** | **18.7 [18.2;19.2]** | **13.3 [13.1;13.5]** | **16.0 [15.3;16.7]** |
| Calcium (mg) | 948 [922;974] | 1014 [979;1050] | 870 [846;893] | 977 [917;1037] |
| Whole Grain (oz) | 0.61 [0.57;0.65] | 0.77 [0.70;0.83] | 0.55 [0.51;0.59] | 0.71 [0.57;0.84] |
| Added Sugar (tspn) | 15.1 [14.5;15.7] | 14.4 [13.6;15.1] | 14.9 [14.2;15.5] | 16.8 [14.8;18.7] |
| Dairy (cup) | 1.50 [1.43;1.57] | 1.59 [1.49;1.69] | 1.42 [1.35;1.48] | 1.60 [1.42;1.78] |
| **Fruits and vegetables inc. fried potatoes (cup)** | **2.38 [2.36;2.40]** | **3.38 [3.29;3.46]** | **1.88 [1.86;1.90]** | **2.75 [2.71;2.79]** |
| **Vegetables inc. fried potatoes (cup)** | **1.53 [1.51;1.55]** | **2.01 [1.94;2.08]** | **1.24 [1.23;1.26]** | **1.63 [1.57;1.68]** |
| **Fruits and Vegetables excl. Fried potatoes (cup)** | **2.23 [2.21;2.25]** | **3.26 [3.17;3.35]** | **1.72 [1.70;1.74]** | **2.60 [2.57;2.64]** |
| **Vegetables excl. Fried potatoes (cup)** | **1.40 [1.38;1.42]** | **1.92 [1.85;1.99]** | **1.10 [1.08;1.11]** | **1.50 [1.44;1.55]** |
| **Fruits (cup)** | **0.81 [0.78;0.84]** | **1.28 [1.20;1.36]** | **0.61 [0.59;0.63]** | **1.13 [1.03;1.24]** |
| Sugar in beverages (tspn) | 6.37 [5.92;6.82] | 5.86 [5.39;6.34] | 6.68 [6.12;7.24] | 7.48 [5.86;9.10] |
